# Therapeutic angiogenesis and tissue revascularization in ischemic vascular disease

**DOI:** 10.1186/s13036-023-00330-2

**Published:** 2023-02-16

**Authors:** Xinyue Chen, Wenlu Yu, Jing Zhang, Xiao Fan, Xiao Liu, Qi Liu, Su Pan, Richard A. F. Dixon, Pengyang Li, Peng Yu, Ao Shi

**Affiliations:** 1 The Second Clinical Medical College of Nanchang University, The Second Affiliated Hospital of Nanchang University, Nanchang, 330006 Jiangxi China; 2 School of Ophthalmology and Optometry of Nanchang University, Nanchang, 330006 China; 3 Department of Anesthesiology, The Second Affiliated Hospital of Nanchang University, Nanchang, 330006 Jiangxi China; 4 Department of Cardiovascular Medicine, The Second Affiliated Hospital of Sun Yat Sen University, Guangzhou, 51000 Guangdong China; 5 Wafic Said Molecular Cardiology Research Laboratory, The Texas Heart Institute, Houston, TX USA; 6 Division of Cardiology, Pauley Heart Center, Virginia Commonwealth University, Richmond, VA USA; 7 Department of Endocrinology and Metabolism, The Second Affiliated Hospital of Nanchang University, Nanchang, Jiangxi Province 330006 China; 8 School of Medicine, St. George University of London, London, UK; 9 School of Medicine, University of Nicosia, Nicosia, Cyprus

**Keywords:** Ischemic vascular disease, Therapeutic angiogenesis, Tissue revascularization, Biological therapy

## Abstract

Ischemic vascular disease is a major healthcare problem. The keys to treatment lie in vascular regeneration and restoration of perfusion. However, current treatments cannot satisfy the need for vascular regeneration to restore blood circulation. As biomedical research has evolved rapidly, a variety of potential alternative therapeutics has been explored widely, such as growth factor-based therapy, cell-based therapy, and material-based therapy including nanomedicine and biomaterials. This review will comprehensively describe the main pathogenesis of vascular injury in ischemic vascular disease, the therapeutic function of the above three treatment strategies, the corresponding potential challenges, and future research directions.

## Background

Ischemic vascular disease (IVD), as one of the deadliest and most disabling diseases, is a condition characterized by the narrowing of blood vessels, which induces the reduction of blood supply and inadequate transport of nutrients and oxygen [1]. Currently, there are three main clinical categories according to the location of the disease: peripheral artery disease (PAD), coronary heart disease (CHD), and carotid artery disease (CAD). PAD is the most common form of IVD, affecting the blood vessels other than the heart and brain. Cold hands or feet, cramping or pain in leg muscles, and reduced or absent arm or leg pulse will occur once the constrictions become severe [2]. CHD is a form of IVD mainly affecting the heart. When the coronary arteries become constricted, the heart muscle undergoes starvation and hypoxia. CAD is caused by stenosis of the carotid arteries. When blood flow to the brain is reduced or blocked by plaque, even for a few minutes, the lack of oxygen may cause damage or even death of brain cells, thus possibly inducing cerebral infarction and threatening life [3]. Emerging studies have declared that vascular damage and ischemia induced by plaque are the core pathogenic mechanisms of IVD [4]. The traditional treatment therapies for IVD include two major categories: surgical procedures and drug treatment. However, many studies have shown these treatments cannot completely reshape the structure of blood vessels and restore blood circulation, thus inadequately satisfying the supply of nutrients and blood [5]. Therefore, alternative therapies targeting angiogenesis and tissue revascularization are needed to advance current IVD treatment plans.

Angiogenesis is defined as the formation of new blood vessels within existing vascular structures, which is required to repair or rebuild tissue and restore tissue perfusion [6]. The process begins with the sprouting of endothelial cells (ECs) in existing capillaries, followed by EC migration, proliferation, and lumen formation. In addition, the expansion of the microvascular system also depends on the intussusception of existing capillaries [7]. In recent years, “therapeutic angiogenesis” has provided new directions in the therapy of IVD: growth factor-based therapy, cell-based therapy, and material-based therapy including nanomedicine and biomaterials.

In this review, we collected updated information from reviews and primary research focusing on the epidemiology, pathogenesis, and treatment of PAD, CHD, and CAD. Moreover, we included preclinical and clinical studies focused on driving angiogenesis through growth factor-based therapy, cell-based therapy, and material-based therapy including nanomedicine and biomaterials from the cell level in the past 5 years. In addition, we examined the underlying biological mechanism, the corresponding potential challenges, and future research directions.

## Vessel damage and tissue ischemia

### Microvascular remodeling

Microvascular remodeling, a pathological process leading to tissue ischemia and hypoxia, is an important driver of IVD [8]. The microcirculation mainly regulates capillary flow and pressure, and optimizes the nutrient and oxygen supply to the surrounding tissue. Microvascular injury triggers a systemic pathophysiological process. Vascular remodeling involves changes in the diameter and/or density of microvessels, regulated primarily by mechanical forces acting on ECs and vascular smooth muscle cells (VSMCs) [9]. The most common mechanisms of vascular injury include vascular endothelial dysfunction, inflammation, and oxidative stress reactions, which eventually lead to chronic organ hypoperfusion [10–12]. Oxidative stress and inflammatory responses caused by excessive production and accumulation of cellular reactive oxygen species (ROS) are believed to be the key mechanisms driving the development of microvascular injury [13]. Ras homolog gene family member A (RhoA)/Rho kinase, is closely associated with ROS synthesis and hypercontraction of VSMCs and regulates the force of smooth muscle contraction by regulating calcium level and phosphorylation of contractile myofibers [14]. In vitro and in vivo studies showed that increased intracellular ROS concentrations promoted the conversion of nitric oxide (NO) into peroxynitrite radicals, resulting in impaired NO-mediated vasodilation and enhanced the production of endothelin-1 (vasoconstrictor agonist) via activation of the RhoA/Rho kinase pathway [14, 15]. A clear understanding of the mechanism of microvascular remodeling is important in exploring the treatment of IVD.

### Peripheral arterial disease (PAD)

PAD is a type of ischemic disease that occurs in major blood vessels other than the central vessels and coronary arteries. Because of plaque formation and atherosclerosis, vascular stenosis and compromised forward blood flow cause various pathological conditions [2]. As a subtype of PAD, central retinal occlusion can lead to retinal ischemia, triggering neuroinflammation and apoptosis, and even causing vision damage and loss. Current therapies, such as intraocular injections of antibodies (anti-VEGF), eye drops, or surgery, cannot fully address these unmet clinical needs, and safer solutions such as retinal-cell-targeting nanoparticles need to be investigated [16]. Critical limb ischemia (CLI) is the end stage of PAD, which often occurs in the lower limbs [17]. The probability of death at 1 year is reported to be as high as 25%, and another 30% of the patients will undergo amputation [18]. The most effective therapy lies in limb perfusion improvement. An increasing number of new technologies are being investigated to provide therapy options for the future, such as growth factors and stem cells [16, 19].

### Coronary heart disease (CHD)

CHD is characterized by coronary artery stenosis and reduced blood supply. About 1 million heart attacks occur each year, and nearly 15% of patients die of a heart attack [20]. If the blood flow is completely blocked, the cardiomyocytes will die, leading to myocardial infarction. If the area of cardiomyocyte necrosis is large, the result is left ventricular remodeling and even the failure of the entire organ in the end. The ideal therapeutic approach in clinical practice is to promote the proliferation of cardiac myocytes, reduce fibrosis, restore cardiac blood supply, and improve cardiac function.

### Carotid artery disease (CAD)

CAD is closely related to carotid atherosclerosis and cardio-cerebrovascular diseases, and reflects the evolution of systemic atherosclerotic diseases. CAD is associated with 15–20% of ischemic strokes [21], which are caused by insufficient blood and oxygen supply to the brain. Ischemia that extends around occluded distal arteries creates lacunar strokes, which may cause recognizable clinical presentations such as pure motor hemiplegia, ataxic hemiparesis, and clumsy hand dysarthria [22]. A short period of insufficient blood supply to the brain may cause a reversible loss of brain tissue, and a cerebral infarction may occur. This scenario is accompanied by loss of neurons and supporting structures as the period of reduced blood supply continues; eventually, ROS accumulate, leading to cell membrane damage and cell lysis [22]. But the current treatment, such as thrombolysis and endovascular stenting, is often limited by the time window of treatment and the possibility of serious bleeding complications [23]. Due to the limited renewal ability and slow conversion of nerve cells, endogenous cells are not sufficient to repair damaged neurons, so cell therapy is of paramount importance. It would provide a much-needed additional approach to the treatment of IVD.

In short, vessel damage and tissue ischemia are vital pathogenic factors for the development of IVD. Therefore, the core of the treatment is vascular regeneration and restoration of tissue perfusion. Next, we will discuss three new potential therapies relevant to angiogenesis and tissue revascularization in IVD.

## Growth factor-based therapy

**Fig. 1:**
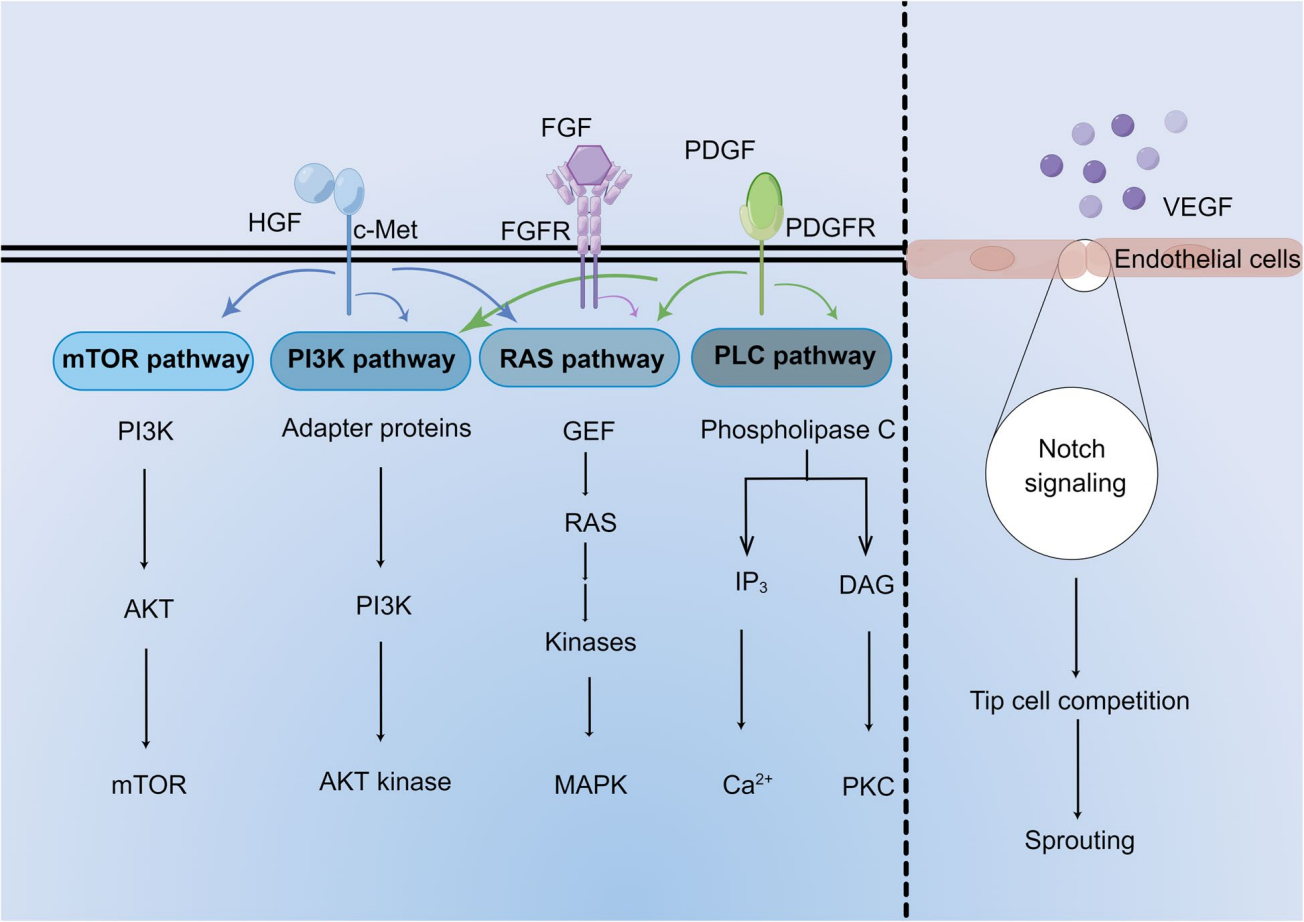
The main therapeutic mechanisms of growth factors in ischemic vascular disease. The mechanism of HGF is mediated through its unique tyrosine kinase receptor (c-Met), which could drive intracellular signaling pathways (including the RAS‐MAPK, PI3K‐protein kinase B, and mTOR pathways). FGF regulates biological functions including cell proliferation, survival, migration, and differentiation by binding and activating FGF receptors via the RAS/MAPK pathway. PDGF gets involved in IVD through the PI3K pathway, the RAS pathway, and the PLC pathway. The induction of endothelial cells called tip, stem, and finger osteoblasts through interactions between VEGF and its receptor (VEGFR1-3) and between Notch and Delta-like nick ligands is essential for the regulation of angiogenesis. c-Met: cellular-mesenchymal epithelial transition factor; DAG: diacylglycerol; FGF: fibroblast growth factor; FGFR: fibroblast growth factor receptor; GEF: GMP exchange factor; HGF: hepatocyte growth factor; IP_3_: inositol triphosphate; IVD: ischemic vascular disease; MAPK: mitogen-activated protein kinase; mTOR: mammalian target of rapamycin; PDGF: platelet-derived growth factor; PDGFR: platelet-derived growth factor receptor; PLC: phospholipase C; PKC: protein kinase C; PI3K: phosphatidylinositide 3-kinases; RAS: rat sarcoma; VEGF: vascular endothelial growth factor; VEGFR: vascular endothelial growth factor receptor. The figure was created by Figdraw (https://www.figdraw.com/)

Growth factors are a class of peptides that regulate cell growth and other cellular functions by binding to corresponding cell membrane receptors [24]. They are secreted by a variety of cells and act on specific target cells to regulate cell division, matrix synthesis, and tissue differentiation [24]. Numerous angiogenic growth factors such as VEGF, fibroblast growth factor (FGF), and hepatocyte growth factor (HGF) have been extensively studied for treating ischemic diseases such as PAD and CHD [25] (Tables 1 and 2 contain a partial list of the preclinical and clinical studies mentioned below). The relevant mechanisms are summarized in Fig. 1.

**Table 1:** A summary of growth factor-based therapeutic preclinical studies in IVD

GF	Disease	Function	Research types	Ref
VEGF	CHD	Increase endothelial cell proliferation and angiogenesis	Animal	[26]
VEGF-A	CHD	Promote angiogenesis through initiating ROS-ER stress-autophagy axis	Cellular and Animal	[27]
HGF	CHD	Anti‐apoptosis	Animal	[28]
HGF	CHD	Anti-apoptosis and promote angiogenesis	Cellular and Animal	[29]
FGF-2	PAD	Promote angiogenesis	Animal	[30]
FGF	CAD	Promote angiogenesis	Animal	[31]
FGF	CHD	Promote angiogenesis	Animal	[32]
PDGF	CHD	Promote angiogenesis	Animal	[33]
PDGF BB	PAD	Promote angiogenesis	Animal	[30]
PDGF-C	PAD	Promote angiogenesis	Animal	[34]
GDF-15	CHD	Anti‐apoptosis and promote angiogenesis	Animal	[35]
IGF-1	CHD	Reduce fibrosis	Animal	[36]

*CHD* Coronary heart disease, *FGF* Fibroblast growth factor, *GDF* Growth differentiation factor, *GF* Growth factor, *HGF* Hepatocyte growth factor, *IGF* Insulin-like growth factors, *IVD* Ischemic vascular disease, *PAD* Peripheral artery disease, *PDGF* Platelet-derived growth factor, *ROS-ER* Reactive oxygen species-endoplasmic reticulum, *VEGF* Vascular endothelial growth factor

**Table 2:** A summary of growth factor-based therapeutic clinical trials in IVD

First author	Phase year	Disease	Treatment	Subject(n) treatment/control	Findings	Ref
Stewart, Duncan J	Phase II 2008	CHD	Intramyocardial VEGF plasmid	48/45	No therapeutic effect	[37]
Richard J Powell	Phase II 2010	CLI	Intramuscular HGF plasmid	21/6	Improved ABI, no change in wound healing, major amputation, or death	[38]
Jill Belch	Phase III 2011	CLI	Intramuscular FGF plasmid	259/266	Failed to reduce amputation or death	[39]
Motoyuki Kumagai	Phase I/IIa 2016	CLI	Intramuscular FGF plasmid incorporated with gelatin hydrogel microspheres	10/0	Provided a safe and effective form of angiogenesis	[40]
Deev, R	Phase IIb/III 2018	CLI	Intramuscular VEGF165 plasmid	36/12	Improved ABI, TcPO_2_ and PWD	[41]
Gu, Y	Phase II 2019	CLI	Intramuscular HGF plasmid	150/50	Completed pain relief and improved the healing of ulcers	[42]
Barć, P	Phase II 2021	CLI	Intramuscular pIRES/VEGF165/HGF	14/14	Increased ABI and vascularization, decreased rest pain	[43]

*ABI* Ankle brachial index, *CHD* Coronary heart disease, *CLI* Critical limb ischemia, *FGF* Fibroblast growth factor, *HGF* Hepatocyte growth factor, *IVD* Ischemic vascular disease, *pIRES* Plasmid internal ribosome entry site, *PWD* Pain-free walking distance, *TcPO2* Transcutaneous oxygen pressure

### Vascular endothelial growth factor (VEGF)

VEGF plays a central regulatory role in the angiogenesis process. The mammalian VEGF family consists of five different polypeptides: VEGF-A, VEGF-B, VEGF-C, VEGF-D, and placental growth factor [25]. VEGF promotes vascular permeability and induces the proliferation of ECs via VEGFR1 and VEGFR2 [25]. In 1996, the first clinical trial of VEGF gene delivery, reported in a 70-year-old patient with PAD, successfully induced angiogenesis [44]. However, the success of only one case did not rule out the role of chance in the experiment. Since then, emerging preclinical and clinical studies have begun to explore VEGF-based therapies in IVD.

In rats with myocardial infarction, Yang, Z., et al. [26] found that VEGF in the infarcted tissue was significantly upregulated at 12 days after infarction when compared with levels at 6 days after infarction. Moreover, EC proliferation and microvessel formation in rat myocardial tissue increased more at 12 days. These observations show that VEGF is important in microvessel formation in the body in ischemic diseases.

Further study of the underlying mechanism of VEGF-driven angiogenesis identified different pathways of this VEGF-related event. Although most studies focused on the classical VEGFR-VEGF pathway, Zou, J., et al. [27] reported that VEGF-driven angiogenesis may also be related to the activation of the ROS-endoplasmic reticulum (ER) stress autophagy axis. They found that VEGF-A increased ROS production in a dose- and time-dependent manner in human umbilical vein ECs, which presented as the corresponding proliferation rates. Similar studies have shown that ROS generated during ischemia are critical for cardioprotective redox signaling that mediates multiple growth-related responses, including angiogenesis [45]. Furthermore, ROS are generally recognized as important inducers of ER stress and autophagy [46]. Similar to compensatory angiogenesis, moderate ER stress and autophagy help maintain homeostasis and tissue development [46]. Lu, Q. et al. [47] reported that autophagy was critical for effective therapeutic angiogenesis in the treatment of IVD. They found that when autophagy was reduced in VSMCs, angiogenesis was inhibited, and infarct size was larger. Furthermore, it has been suggested that maintaining or enhancing autophagy may be an innovative strategy to improve the efficacy of therapeutic angiogenesis [47]. Therefore, it is worthwhile to explore the balance between VEGF-A, ROS, and ER stress for optimal compensatory angiogenesis.

Despite the promising results seen in preclinical outcomes, clinical outcomes have not been consistent. In a 5-year follow-up study in PAD patients, Deev, R. et al. found that VEGF165 plasmid therapy was safe and effective [41]. To our knowledge, this is one of the longest-term studies designed to assess the safety and efficacy of growth factor gene therapy in IVD. However, their outcome measures such as pain-free walking distance have a high risk of detection bias, which would affect the reliability of the results. Interestingly, in terms of clinical application in the treatment of CHD, VEGF has no therapeutic effect. Jens Kastrup et al. found that VEGF gene transfer did not significantly improve myocardial perfusion in patients with ischemic heart disease [48]. Additionally, in the NORTHERN trial of Stewart, Duncan J., et al. [37], patients with CHD received an endocardial injection of 2,000 µg of VEGF plasmid; however, the treatment did not improve disease status. First, we cannot evaluate the transfection rate of VEGF in human myocardial tissue. A recent preclinical study showed that inflammatory responses attenuated the therapeutic effect of VEGF gene delivery [49], which would significantly affect outcomes. Second, we speculate that the therapeutic effect of VEGF may be related to the diseased tissue itself, which deserves further exploration. Third, emerging studies demonstrated that VEGF stimulated the growth of malfunctioning and leaky vasculature, which would not support normal perfusion requirements [50].

### Hepatocyte growth factor (HGF)

HGF is a multifunctional cytokine with critical roles in proliferation, survival, motility, and morphogenesis [51]. The biological function of HGF is mediated through its unique tyrosine kinase receptor cellular-mesenchymal epithelial transition factor (c-Met) that could drive intracellular signaling pathways (including the RAS‐Mitogen-activated protein kinases (MAPK), phosphoinositide -3 kinase (PI3K) ‐protein kinase B, and mammalian target of rapamycin (mTOR) pathways) [51]. The activation of HGF/c-Met signaling can promote angiogenesis, inhibit apoptosis, regulate inflammation, and stimulate tissue regeneration [51]. In a study in rats, Liu et al. [29] ligated the left anterior descending coronary artery and immediately injected a total of 50 µl adenovirus (Ad)-HGF into the myocardium in the vicinity of the ischemic region of the left ventricular wall. They found that Ad-HGF treatment enhanced levels of anti-apoptotic proteins (including B-cell lymphoma-2 and B-cell lymphoma-extra-large) and simultaneously decreased levels of pro-apoptotic Bax. Moreover, SU11274, a specific inhibitor of the c-Met receptor of HGF, could effectively block the inhibitory effect of Ad-HGF on apoptosis [29]. In another animal study, Zhang, Z., et al. [28] reported similar results. However, Zhang, Z., et al. used a diabetic rat model to observe the therapeutic effect of HGF on cardiac function in myocardial infarction. They revealed that by further enhancing the activation of the HGF/c-Met pathway, HGF significantly alleviated apoptosis of cardiomyocytes after acute myocardial infarction in diabetic rats and improved cardiac function [28]. Rong, SL et al. [52]., also found that HGF gene transfection improved left ventricular remodeling after myocardial infarction in rats, which was associated with induction of angiogenesis, inhibition of cardiomyocyte apoptosis, and reduction of pro-inflammatory cytokine expression.

The success of preclinical experiments led to clinical trials. A clinical trial from China explored HGF function in CHD therapy in 21 patients with severe CHD [53]. Of the study group, 11 patients received both a stent and administration of Ad-HGF (intracoronary drug delivery), and the remaining 10 patients received a stent alone and served as the control group. Before and then 6 and 24 h, 3 and 6 days, and 2 weeks after treatment, researchers collected blood samples from the femoral vein. The data showed that Ad-HGF in patients with CHD resulted in high levels of HGF gene expression, as well as its receptor c-Met. The promotion of angiogenesis and heart function was observed in patients treated with Ad-HGF [53]. However, their short observation time and the small sample size hinder the extrapolation of these findings to the entire CHD population. A phase II clinical trial for CLI treatment also confirmed the therapeutic effect of HGF [42]. In this trial, 150 patients with CLI received an intramuscular injection of NL003 (a plasmid DNA encoding a genomic cDNA hybrid human HGF sequence). Unlike other clinical trials, this trial randomly divided 150 patients into three groups of low-dose (12 mg), medium-dose (18 mg), and high-dose (24 mg) injections. Although CLI conditions improved in all three groups (healing of leg ulcers and complete pain relief), improvement was significantly better in the high-dose group than in the other two groups. This determined the appropriate NL003 injection dose for phase III clinical trials. However, in a clinical trial involving 48 patients with CLI, Powell RJ, et al. [38] found that intramuscular injection of HGF significantly improved the ankle-brachial index (ABI) at 6 months when compared with baseline but did not result in statistically significant differences in the rates of complete wound healing, major amputation, or death at 6 months compared with placebo. Given the wide variability in the degree of tissue necrosis present in the CLI patient population (variations in tissue loss at presentation), the differing results seen in clinical trials may be related to patient characteristics, such as the wide variability in the degree of tissue necrosis found in CLI. Moreover, the dose and frequency of administration are also important influencing factors. However, we can confirm that HGF gene therapy is safe and well-tolerated. Preliminary trials using HGF gene therapy are promising and warrant the conduct of larger clinical studies. In the clinical trial of Barć P, et al. [43], compared with the control group, CLI patients who received VEGF/HGF bicistronic gene therapy had better outcomes in VEGF level and functional recovery. They observed that serum VEGF levels and the ankle-brachial index were higher at 90 days after plasmid administration than before plasmid administration, and the pain level was reduced significantly. Moreover, computed tomography angiography showed significant improvement in vascularization [43]. Due to financial constraints, this study measured VEGF plasma levels only in the treatment group, which prevented the evaluation of the expression of HGF and the expression of the growth factors in the control patients. However, the idea of co-treatment of multiple growth factors may be a potential treatment method in IVD.

### Fibroblast growth factor (FGF)

The FGF family comprises 22 members of whom FGF-1 and FGF-2 have angiogenic potential. Emerging studies have investigated the important therapeutic effect of FGF in IVD [54]. FGF binds to its receptors through the RAS/MAPK pathway and regulates biological functions such as cell proliferation, survival, migration, and differentiation [54]. In several animal studies, FGF-2 not only improved myocardial perfusion and protected against ischemia–reperfusion damage to the heart but also enhanced capillary growth in hindlimb ischemia models [30, 55]. Pang, Q., et al. revealed that the protective effects of basic fibroblast growth factor (bFGF) in focal cerebral ischemia in rats are relevant to angiogenesis and neurologic functional recovery [31]. In the treatment of CHD, Reigstad, L.J., et al. [32] reported that bFGF promoted angiogenesis. They also observed higher systolic function and lower left ventricular end-diastolic pressure in rats treated with bFGF [32]. In addition to rats, rabbits have been used to study the effect of FGF on IVD. In a rabbit hindlimb ischemia model, Li, J. et al. found that naked plasmid FGF-2 promoted capillary growth, collateral formation, and popliteal blood flow [30]. Successful studies in multiple animal models promote the progress of clinical research.

In clinical trials, FGF shows potentials in treating IVD. Laham RJ et al. explored the safety, tolerability, and preliminary efficacy of intracoronary FGF-2 by administering escalating doses of FGF-2 in 52 patients with CHD [56]. They found that the treatment was safe and reduced the extent of the ischemic area. In this study, proteins rather than plasmids were directly injected. A pertinent question is how did FGF-2, which has a relatively short plasma half-life, promote the relatively long-time formation of vascular collaterals after entering the human body? One contributing factor may be that the transient accumulation of FGF-2 in ischemic myocardium initiates a self-amplifying cascade that includes monocyte/macrophage influx and endothelial adhesion [57], which induces a relatively long-term effect of FGF-2. In a phase II double-blind, randomized, placebo-controlled, multicenter study that enrolled 125 patients with CLI, Sigrid Nikol et al. [58] gave the treatment group an intramuscular injection of NV1FGF (a plasmid-based angiogenic gene delivery system for local expression of FGF-1). They [58] found that NV1FGF treatment failed to improve ulcer healing but reduced the risk of major amputation or death at 1 year when compared with placebo. However, in a phase 3 clinical trial published in Lancet [39], 259 patients with CLI received NV1FGF treatment in the diseased leg. The results showed that NV1FGF treatment did not reduce amputation or death at 1 year. In comparing the two clinical trials, we speculate that the lack of any significant effect on ulcer healing may be due to the large heterogeneity and severity of baseline skin lesions in CLI patients. Some patients have complete necrosis of distal tissue, which fails to heal even with increased blood flow. The difference in the effect of amputation and mortality may be related to the design of the experiment itself and the patients’ medical conditions.

In a recent clinical trial, Kumagai, M. et al. [40] treated 10 CLI patients with gelatin hydrogel microspheres incorporated with bFGF intramuscularly at 4 weeks and 24 weeks. They analyzed the safety of this approach and the transcutaneous oxygen pressure in patients and found that sustained release of bFGF may provide a safe and effective form of angiogenesis in patients with CLI. Considering that the short half-life of a single injection of growth factor protein is not enough to achieve an effective dose and that repeated and prolonged injections will cause systemic toxicity, gelatin hydrogel can effectively prolong the action time and improve the efficiency of vascular growth factors. This combination approach is further addressed in the biomaterial section.

### Platelet-derived growth factor (PDGF)

PDGF consists of four polypeptide chains, namely, PDGF-A, PDGF-B, PDGF-C, and PDGF-D, which form four homodimers including PDGF-AA, PDGF-BB, PDGF-CC, and PDGF-DD and one heterodimer, PDGF-AB [59]. PDGF plays vital roles in embryogenesis, organogenesis, and the formation of blood vessels [59]. In rats with occlusion of the left anterior descending coronary artery, injecting 1 × 10^11^ copies of recombinant adeno-associated virus 9-mediated vector genomes to overexpress PDGF-A in the injured heart promoted angiogenesis and cardiac repair [33]. In a hindlimb ischemia model in diabetic mice [34], gain of function and loss of function experiments were performed to understand the angiogenic properties of PDGF-C. Intramuscular injection of PDFG-C expression vector accelerated blood perfusion in ischemic limbs, whereas PDFG-C knock-out mice showed reduced blood flow recovery. Moreover, the impaired perfusion recovery in the ischemic legs of diabetic mice was positively associated with reduced PDGF-C expression in ischemic tissues, and overexpression of PDGF-C in the ischemic legs ameliorated the flow deficits. This study highlighted a potential role of PDGF-C in treating leg ischemia in diabetes.

In a clinical trial of patients with ischemic stroke, Moniche, F. et al. [60] found that intraarterial bone marrow mononuclear cell transplantation in patients with ischemic stroke increased serum PDGF-BB levels; higher PDGF-BB levels were associated with better functional outcomes during follow-up at 90 days. This was related to the effects of PDGF-BB in improving neural stem cell migration, angiogenesis, and regeneration of damaged axons after stroke. Furthermore, considering that intracoronary infusion of bone marrow mononuclear cells is known to improve left ventricular function after acute myocardial infarction [61], Mahan Shahrivari et al. [62] found that PDGF-BB may promote the recovery of myocardial function in patients with acute myocardial infarction by enhancing the function of bone marrow mononuclear cells. Notably, research has focused on preventing restenosis after percutaneous transluminal coronary angioplasty by inhibiting PDGF from binding to prevent its proliferative and migratory effect on VSMCs located on the inside of blood vessels [63]. Given the multifaceted functions of PDGF in vascularization in numerous IVD animal models, more detailed mechanistic studies are warranted to determine how to promote neovascularization while minimizing vascular dysfunction.

In addition to the above-mentioned growth factors, several others, such as growth-differentiation factor-15 [35] and insulin-like growth factor-1 [36], may have the potential to treat IVD. The various growth factors are potential opportunities to be explored for IVD therapy. Preclinical experiments have shown a therapeutic role for these factors in IVD, but their clinical application is fraught with challenges. The main challenge is that growth factors cannot be stably and continuously expressed and functional in vivo. Peptide growth factors rapidly degrade (e.g., the half‐life of VEGF in vivo is about 30 min), which makes the therapy ineffective [64]. The small peptide, prominin-1-derived peptide (PR1P), is derived from the extracellular region of prominin-1 and binds to VEGF, thus avoiding proteolytic degradation [65]. Adini, A. et al. found that systemic delivery of PR1P upregulated endogenous VEGF within the ischemic myocardium, promoting ischemic tissue recovery in rats with CHD [66]. Another important area of study is the use of biomaterials with protective characteristics to target the transport of growth factors. However, there are still a few issues to consider. First, what is the appropriate dose for injections? Low doses are safe but ineffective, whereas high doses may not be safe. Dose-dependent preclinical studies and clinical trials will be helpful in optimizing growth factor-based therapy. Another issue to address is the systemic toxicity of some growth factors such as VEGF [67]. Growth factor cocktails may provide synergistic effects that may minimize toxicity. Additionally, nanomedicine and biomaterials have achieved promising results in IVD treatment, so combining them with growth factors may address some existing limitations.

## Cell therapy

**Fig. 2:**
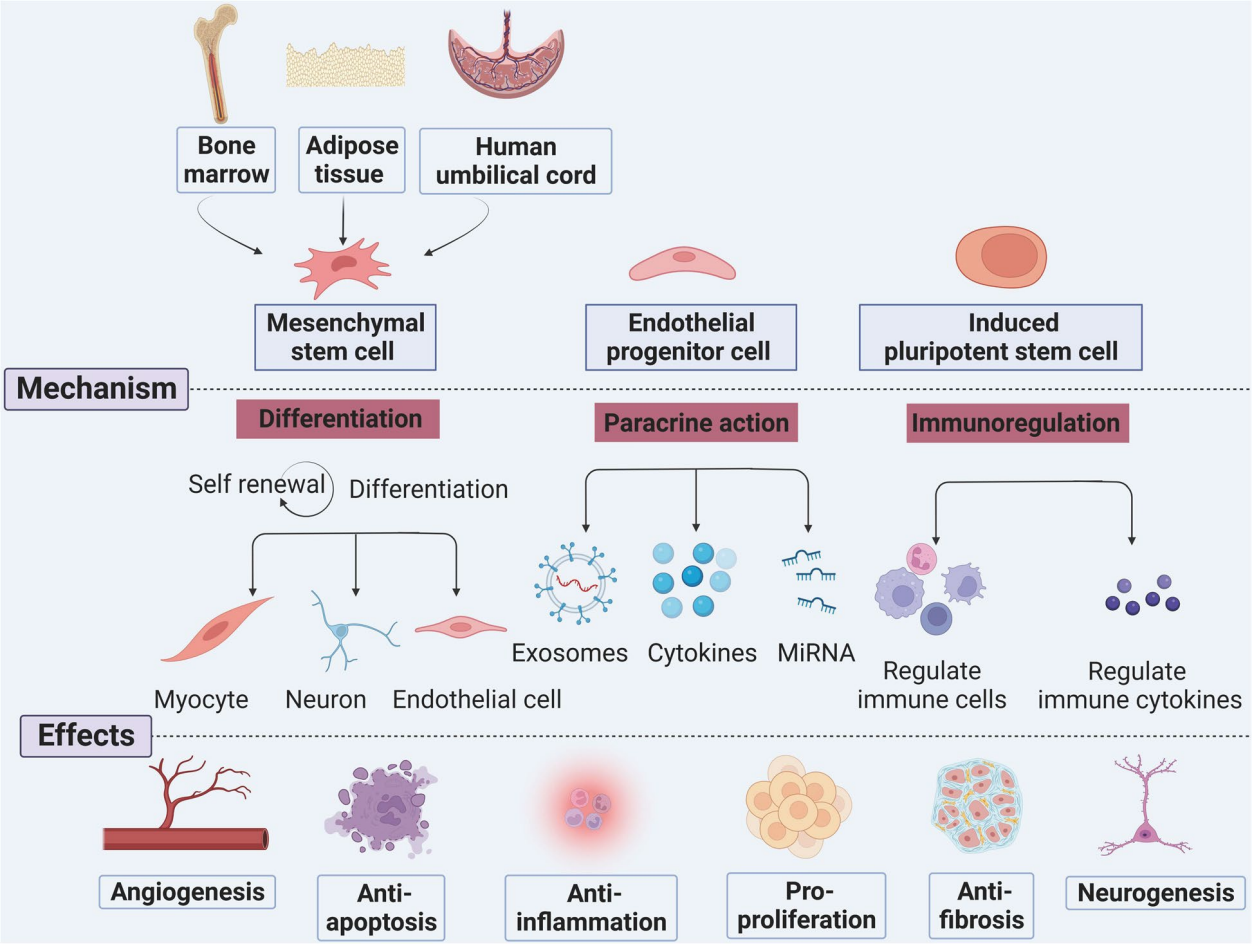
The main therapeutic mechanism of stem cells in ischemic vascular disease. Mesenchymal stem cells, endothelial progenitor cells, and induced pluripotent stem cells are the main stem cells used to treat ischemic vascular disease. These cells mainly exert their angiogenesis, anti-apoptosis, anti-inflammation, pro-proliferation, anti-fibrosis, and neurogenesis function through differentiation, paracrine effects, and immunoregulation. The figure was constructed with BioRender (https://biorender.com/)

Recently, cell therapy has attracted attention for the treatment of IVD (Fig. 2). Stem cell therapy has become a major force for promoting cardiac regeneration and for treating ischemic stroke and CLI [68]. Stem cells, as a self-renewing, high proliferation, and multiple differentiated cell population, have two distinctive features: the ability to differentiate into multiple mature cell types and the ability to replenish the stem cell pool simultaneously [69]; these features make cell therapy a promising approach for treating ischemic disease. Several of the stem cells that play indispensable roles in IVD will be described in detail. (Table 3 contains a partial list of the studies.)

**Table 3:** A summary of stem cell-based therapeutic preclinical studies in IVD

Cells	Disease	Function	Research types	Ref
BMSCs	CHD	Modulate immune response	Animal	[70]
	PAD	Perfusion recovery Running capability increased	Animal	[71]
ADSCs	PAD	Muscle function improved Perfusion increased Inflammatory infiltrate reduced	Animal	[72]
	CAD	Angiogenesis Neurogenesis	Animal	[73]
HUCMSCs	CHD	Improve cardiac systolic function Reduce cardiac fibrosis Suppress cell apoptosis Promote cell proliferation Angiogenesis	Animal	[74]
	PAD	Promote angiogenesis Improve blood perfusion	Animal	[27]
	CAD	Suppress inflammatory responses and neuronal apoptosis	Animal	[75]
	CAD	Decrease brain injury loss Reduce neurological function deficits Increase VEGF expression	Animal	[76]
EPCs	CAD	Angiogenesis	Animal	[77]
	PAD	Angiogenesis	Animal	[78]
iPSCs	CHD	Improve cardiac function	Animal	[79]
	CHD	Inhibit ventricular remodeling	Animal	[80]
	CHD	Improve cardiac function	Animal	[81, 82]

*ADSCs* Adipose tissue derived stem cells, *CAD* Carotid artery disease, *CHD* Coronary heart disease, *EPCs* Endothelial progenitor cells, *HUCMSCs* Human umbilical cord mesenchymal stem cells, *iPSCs* Induced pluripotent stem cells, *IVD* Ischemic vascular disease, *MSCs* Mesenchymal stem cells, *PAD* Peripheral artery disease

### Mesenchymal stem cells (MSCs)

MSCs are multipotent stem cells originating from tissue such as umbilical cord, fat, and bone marrow [83]. They can differentiate into various terminal cell types, including, adipocytes, ECs, cardiomyocytes, and skeletal muscle cells [84]. MSCs are easily obtained from multiple tissues and have low immunogenicity, which allows for allotransplantation [84]. Studies have shown that mechanotransduction such as fluid flow, hydrostatic pressure, compression, and tensile stress can help MSCs to differentiate into their designated lineages [85]. The main mechanisms for treating ischemic diseases are differentiation to ECs, immunoregulation, and paracrine action [84]. The paracrine effect refers to the communication between adjacent cells mediated by growth factors, cytokines, and other regulatory molecules. MSCs can secrete a variety of cytokines, which is important in IVD therapy [86].

#### Bone marrow mesenchymal stem cells (BMSCs)

Bone marrow is one of the most common sources of MSCs [84]. Quevedo, H.C., et al. found that when mice with myocardial infarction were treated with BMSCs, the infiltration rate of granulocytes was reduced in the damaged tissue [70]. Notably, it has been reported that gene modification of BMSCs can enhance the cells’ paracrine function and achieve targeted tissue regeneration. The phosphoinositide 3-kinase/kinase B (PI3K/AKT) signaling pathway phosphorylates its target proteins through various downstream pathways and plays a role in the survival, proliferation, migration, and angiogenesis induction ability of MSCs, thus improving the prognosis of myocardial infarction [87]. In in vitro cell culture, some stimulators of the PI3K/AKT pathway such as transforming growth factor-β1 and prostaglandin E(2) have been used to stimulate MSC migration [88, 89]. Moreover, stromal-derived factor-1 (SDF-1), as a stimulator of the PI3K/AKT pathway, plays a role in mediating both migration and angiogenesis induction ability [90]. Tang, J., et al. demonstrated that rats with myocardial infarction that received Ad-SDF-MSC had increased vascular density and decreased infarct volume [91, 92]. This suggests that activation of the PI3K/AKT signaling pathway can improve MSC performance in tissue engineering, and its role in the treatment of acute myocardial infarction deserves further investigation.

BMSCs also play a therapeutic role in ischemic stroke via their paracrine actions. Studies have shown that BMSCs can transform microglia from a attacking to a neuroprotective phenotype by releasing the chemokine fractalkine [93]. Stem cell therapy can also be combined with other treatments to enhance the therapeutic effect. Deng, Y., et al. discovered a novel triple-catalytic enzyme that can stably produce prostacyclin (PGI2-hMSCs); this enzyme promoted blood perfusion and enhanced the recovery of motor ability in mice with hindlimb ischemia through paracrine effects [71].

In a recent randomized clinical trial of the efficacy of BMSCs on ischemic stroke, 16 patients received intravenous BMSCs, and 15 patients served as the control group. The median duration of rehabilitation was shorter in the treated group than in the non-treated group. No significant difference in tumorigenesis, pro-inflammatory effects, or other adverse events was observed between the two groups [94]. These exciting results suggest that intravenous delivery of BMSCs might be safe and feasible for post-stroke patients. However, purity, consistency, low cell yields, and increased costs in manufacturing are challenges for their clinical application [84]. In addition, transplanting cells in the setting of an acute myocardial infarction may be harmful to the cells because of the hypoxia of damaged tissue and the high concentration of free radicals. Another major challenge is the potential intercellular interaction between ischemic cardiomyocytes and injected cells, which reduces transplantation efficiency. Different methods of preparing BMSCs may also affect the therapeutic properties of the cells. Lee, J., et al. demonstrated that human MSCs can be mechanically and pharmacologically conditioned to enhance vascular regeneration in vivo [95]. Future studies are warranted to address these challenges in translating cell therapy into clinical use.

#### Adipose tissue-derived stem cells (ADSCs)

ADSCs are stem cells obtained from subcutaneous adipose tissue that can differentiate into cardiomyocytes, neurons, and hepatocytes under specific conditions. Some experiments have shown that the main mechanism of ADSCs in treating CHD is to promote angiogenesis by paracrine action, which can improve cardiac function and prevent myocardial remodeling. Allogeneic ADSCs injected into the femoral vein of rats with middle cerebral artery occlusion were shown to protect brain function, reduce brain cell death, and promote angiogenesis and neurogenesis by increasing VEGF secretion [73]. Furthermore, a combination of allogeneic ADSCs and macrophages injected into the ischemic gastrocnemius muscle in rats with PAD can reduce the level of inflammation, promote recovery of muscle function and histopathologic effects, and improve limb ischemia–reperfusion [72]. The studies described above used allotransplantation, but whether this approach will lead to rejection requires further examination. However, a study conducted by Wang, Y., et al. showed that the ex-*vivo* pretreatment of allografts using ADSCs may function as an important adjunctive therapy for inducing immunotolerance in vascularized composite tissue allotransplantation [96]. Notably, Min KH. et al. [97] found that ADSCs with low-level laser therapy (LLLT) showed stronger proliferation ability in vitro. Moreover, the proliferation ability of ADSCs was enhanced in vivo both when ADSCs with LLLT were transplanted into normal rats and when ADSCs without LLLT were transplanted into rats that were irradiated with low-intensity laser after transplantation. These results revealed that light intensity might be a critical therapeutic effect of ADSCs on IVD and deserves further study.

Although both BMSCs and ADSCs can differentiate into several cell types, ADSCs demonstrated superior properties over BMSCs, such as relatively higher cell densities and higher proliferation rates [98]. Moreover, ADSCs have better tolerance to hypoxia, lower immunogenicity, and higher immunosuppression capacity, but lower transcriptomic heterogeneity [99]. Furthermore, the technique to obtain ADSCs is minimally invasive [100]. More preclinical and clinical studies may be useful in identifying the optimal therapeutic applications for each cell type.

#### Human umbilical cord mesenchymal stem cells (HUCMSCs)

Compared with other stem cells, HUCMSCs have many advantages such as low immunogenicity, non-invasive harvest procedure, and ease of expansion in vitro [101]. Zhao, Y., et al. discovered that the secretomes of HUCMSCs can improve cardiac systolic function, reduce cardiac fibrosis, suppress cell apoptosis, and promote cell proliferation and angiogenesis in rats with acute myocardial infarction [74]. In a PAD model, Wang, Z., et al. injected BMSCs/ HUCMSCs into the ischemic hind limb of six-week-old mice and found that the cells promoted angiogenesis better than BMSCs did, and HUCMSCs exerted a stronger effect on EC proliferation and tube formation compared with BMSCs in vitro. Blood perfusion in the HUCMSC group was better than that in the BMSC group in vivo [27]. In treating ischemic stroke by injecting HUCMSCs intravenously into rabbits with middle cerebral artery occlusion, Zhu, Y., et al. found that the inflammatory response and neuronal apoptosis were suppressed after HUCMSC transplantation [75]. Similarly, Liao et al. reported that 24 h after occluding the middle cerebral artery in rats, the intracerebral transplantation of HUCMSCs alleviated brain injury loss and neurological function deficits, and increased VEGF expression [76]. The time and method of injection and the cell dosages differ in the two studies described above; therefore, the optimal therapeutic application of cell-based therapy with HUCMSCs remains to be determined.

### Endothelial progenitor cells (EPCs)

EPCs are the precursors of vascular ECs and are characterized by the expression of endothelial surface markers. The EPCs release growth factors and cytokines, which can promote neovascularization in response to tissue ischemia and vascular injury, suggesting angiogenic activity both in vivo and in vitro [102]. EPCs can be used as an appropriate source of vascularization for endovascular prostheses or other artificial materials for alternative therapies and regenerative medicine [103]. The mitogen-activated protein kinases/extracellular signal-regulated kinase (MAPK/ERK) pathway is reported to be associated with cell proliferation, differentiation, migration, senescence, and apoptosis [104]. Studies have clarified that this pathway is also related to the differentiation of EPCs along with their in vitro functions [105]. The MAPK/ERK pathway provides a new therapeutic target for IVD. In animal models of ischemic brain injury, transplantation of EPCs enhanced angiogenesis and improved the perfusion and function of ischemic tissue [77]. Moreover, Kong, L., et al. discovered that a microgravity environment can enhance the angiogenic properties of EPCs' paracrine signals, which promoted angiogenesis in the fractured area in a rat tibial fracture model and accelerated fracture healing [78]. This study shows that more advanced therapies are waiting to be discovered through biomechanical stimulation. More recently, coculture of MSCs and EPCs demonstrated a synergistic effect in angiogenesis [106], thus suggesting more avenues for future treatment.

However, there are certain limitations to cell resources. EPCs are relatively rare (only 0.0084% in the peripheral blood) and thus challenging to obtain for cell therapy. Moreover, the survival rate of transplanted EPCs is low, which may be related to the poor growth environment in ischemic tissues [107]. Recently, cyclic uniaxial mechanical strain was shown to stimulate the differentiation of EPCs into mesenchymal-like cells, which expands the differentiating range of EPCs [108].

### Induced pluripotent stem cells (iPSCs)

iPSCs are somatic cells that are reprogrammed to the pluripotent state through gene activation of key transcription factors [109]. The main advantages of these cells are their almost unlimited self-renewal ability and pluripotency. When considering myocardial tissue, iPSCs can differentiate into various cardiac lineages, including cardiomyocytes, smooth muscle cells, ECs, and cardiac progenitors [110]. Since iPSCs can be obtained from patients’ somatic cells, they have a low risk of immune rejection [111]. However, the risk of teratoma formation has been reported, which should be further investigated to minimize this adverse event [111].

iPSCs have been shown to have therapeutic potential for myocardial infarction in animal models and in clinical trials. Nelson et al. [79] showed that iPSCs improved cardiac function in mice with coronary artery ligation. Histological analysis showed the presence of iPSC-derived cardiomyocytes, VSMCs, and ECs in the damaged myocardium [79]. Kawamura, M., et al. implanted iPSC-derived cardiomyocytes (iPSC-CMs) into infarcted heart muscle in pigs and found improved left ventricular ejection fraction and reduced left ventricular remodeling [80]. Studies have shown improved cardiac contractile function after intracardial injection of allogeneic iPSC-CMs in monkeys with CHD [81]. Notably, in the two studies described above, iPSC-CM sheets and direct cell injections were used, respectively, to transplant the cells. iPSC-CM sheets are a new approach that can achieve the effect of transplanting many cells at one time. In the treatment of CHD, in addition to single iPSC injections, a combination cell injection approach has been explored. By injecting human iPSC-CMs into the myocardium and implanting human-MSCs into the epicardium in rats with myocardial infarction, Park, S.J., et al. found that fibrosis was reduced, and angiogenesis and myocardial repair were promoted [82]. In their rat model, human iPSC-CMs were injected at two different sites in the border zone of the infarcted myocardium immediately after MI induction by LAD ligation, and a human-MSC-loaded patch was implanted directly into the epicardium by using two sutures. The combination of iPSC-CMs and human-MSCs helped improve the survival rate of iPSC-CMs, but simplifying the surgical protocol is a challenge for future studies.

Stem cells are of great value in treating ischemic diseases; they can be obtained from a variety of tissues, and have a high proliferation rate and the ability to renew infinitely. For treating IVD, stem cells promote angiogenesis, neurogenesis, and improvement of neurological/cardiac function through differentiation, paracrine action, and immunoregulation. Direct injections of stem cells have been shown to promote angiogenesis; however, another potential research approach is to study the regulation of the stem cell niche itself. In a mouse model of hindlimb ischemia, Liu, Q. et al. [112] found that inhibiting the Hippo pathway (a growth-inhibiting signaling pathway) can effectively provide structural support for the muscle stem cell niche, thereby promoting skeletal muscle regeneration. Notably, many clinical trials (Table 4) have shown that stem cell therapy is safe and feasible. Different types of MSCs, such as BMSCs, HUCMSCs, and ADSCs, have been studied in treating various IVDs, including CAD and CHD. These studies have shown that MSCs promoted morphological and functional improvement, including better tissue viability, improved cardiac function, and reduced symptoms.

**Table 4:** A summary of stem cell-based therapeutic clinical trials in IVD

First author	Phase year	Disease	Treatment	Subject(n) treatment/control	Findings	Ref
Gao, L.R	Phase I/II 2015	CHD	Intracoronary infusion of HUCMSCs	58/58	Myocardial viability/perfusion increase LVEF increase Proven safety	[113]
Qayyum, A.A	Phase II 2019	CHD	Intramyocardial injections of ADSCs	40/20	Low incidence of angina Chest discomfort reduced NYHA classification reduced	[114]
Mathiasen, A.B	Phase II 2019	CHD	Intramyocardial injections of BMSCs	40/20	LVESV reduced Cardiac pump function and myocardial mass improved Myocardial scar tissue reduced Fewer admissions for angina symptoms	[115]
Jaillard, A	Phase I 2020	CAD	Intravenous autologous MSC treatment	20/11	Proven safety and feasibility	[94]

*ADSCs* Adipose tissue-derived stem cells, *BMSCs* Bone marrow mesenchymal stem cells, *CAD* Carotid artery disease, *CHD* Coronary heart disease, *CLI* Critical limb ischemia, *HUCMSCs* Human umbilical cord mesenchymal stem cells, *IVD* Ischemic vascular disease, *LVEF* Left ventricular ejection fraction, *LVESV* Left ventricular end-systolic volume, *MSCs* Mesenchymal stem cells, *NYHA* New York Heart Association

Despite the safety and feasibility profile of cell therapy seen in these trials, more real-world data are needed to prove the efficacy of this approach and to address the shortcomings identified in the clinical trials. Using positron emission tomography (PET), Gao, L.R., et al. [113] identified an absolute increase in myocardial viability after stem cell treatment. However, PET is a relatively advanced technology, is not commonly used to detect myocardial viability, and is not available for use at all centers. Thus, the experimental method of Gao, LR, et al. is not widely applicable and is not easily reproducible. More general methods are worth studying in clinical trials. Additionally, well-designed clinical trials are important. The number and sex of the patients in the clinical trials can affect the accuracy of the results. For example, Qayyum, A.A., et al. [114] enrolled only men in the placebo group of their clinical trial, which can skew the results. Mathiasen, A.B., et al. [115] enrolled a total of only 60 patients in their trial. Larger studies are needed. The field of cell therapy is transitioning from the use of autologous stem cell transplantation to allotransplantation; this change may help to reduce possible negative effects of the patient's own factors and characteristics on the cells [98]. For example, the population doubling number of MSCs from older donors was lower than that from younger patients, and the ability to form adherent colonies declined with age [116]. Renal failure and anemia can impair the angiogenic function of bone marrow cells [117]. Moreover, more research is required to identify which cell types are more suitable for treating specific IVDs. Notably, the injection of high doses of cells into arteries may cause adverse effects, such as recurrent stroke [94].

Other issues must also be considered. How can we ensure purity in cell products and increase cell yield and survival rate during cell extraction? A more standardized in vitro protocol should be developed to examine and assess cellular characteristics in cell products. Moreover, high-throughput screening on stem cells should be performed to compare extraction protocols. How can we improve the limited differentiation ability of cells? The culture environment, cell resources, and donor characteristics should all be considered. Finally, how can we avoid the formation of teratomas? Further in vitro studies on teratoma formation are warranted.

## Therapy based on nanomedicine and biomaterials

### Nanomedicine

#### Exosomes

Exosomes, a subtype of extracellular vesicles, arise from the membranes of multivesicular bodies and can transmit multiple biological molecules (including proteins, mRNA, and microRNA), thus regulating intercellular communication in pathological or physiological states [118]. Xu, R. et al. [119] treated ischemic stroke in mice with MSC-derived exosomes and found that the microvessel density along the ischemic border zone was significantly improved and the inflammation caused by ischemic stroke was relieved. However, most of the injected MSC-derived exosomes were trapped in the liver. Thus, improving the targeting of exosome delivery is crucial. Cai, G. et al. [120] also found similar therapeutic effects in mouse studies. Moreover, they established a therapeutic role for miR-542-3p in MSC-derived exosomes for IVD, suggesting that certain miRNAs may be crucial in treating IVD and that exosomes have great potential as nanocarriers for drug delivery. In addition, exosomes can transfer bioactive proteins that contribute to tissue revascularization. A recent study demonstrated the potential of an exosome transferring VEGFR2 to stimulate local angiogenesis in a PAD rat model [121].

There are currently no clinical studies of exosomes for treating IVD. Several factors may contribute to this lack of studies. First, there is no uniform method for isolating and purifying exosomes. Second, despite good results in exosome studies in animal models, there are no standard dosage regimens, which remains an uncertain factor that could affect treatment outcomes in clinical trials. Third, the homing efficiency of exosomes from different sources is unknown.

#### Nanoparticles (NPs)

Considering the vital role of exosomes in IVD, Liu, S., et al. explored the function of synthetic magnetic NPs to collect exosomes from the blood circulation [122]. They found that improved angiogenesis and heart function in infarcted heart tissue was induced by the local accumulation of exosomes collected from the circulation using magnetic NPs. The magnetic NP consists of a Fe_3_O_4_ core and a silica shell that is covered with poly (ethylene glycol) conjugated through hydrazone bonds to two types of antibodies, which bind either to CD63 antigens on the surface of exosomes or to myosin-light-chain surface markers on injured cardiomyocytes. In rabbit and rat models of myocardial infarction, the magnetic-guided captured CD63-expressing exosomes accumulated in infarcted tissue, thus promoting angiogenesis and improving recovery [122]. Designing NPs that aggregate exosomes with therapeutic effects may help address the homing efficiency of exosomes. In addition, Florian, A. et al. [123] found that ultrasmall superparamagnetic iron-oxide improved infarct healing and left ventricular remodeling in CHD patients. Although promising, these results were obtained from a small sample size. Larger clinical trials are needed for confirmation. However, NP-mediated antioxidative therapy has shown promise in treating IVD. Jung, E., et al. [124] developed indocyanine green-loaded boronated maltodextrin (ICG-BM) NPs for PAD imaging and therapy. In a mouse model of hindlimb ischemia, they reported that ICG-BM NPs strikingly decreased the level of overproduced H_2_O_2_ and had highly potent anti-inflammatory and proangiogenic functions. Their findings suggest that ICG-BM NPs might exert therapeutic actions by scavenging overexpressed H_2_O_2_, relieving inflammation, and promoting angiogenesis in PAD.

Clinical trials of NPs have recently been launched. NP-mediated drug delivery systems targeting ECs may be an innovative therapeutic strategy. Statins are widely used as cholesterol-lowering drugs [125]. But recent studies have found that they can also increase angiogenic activity [126], suggesting that statins may be suitable candidates for NP-mediated drug delivery treatment of IVD. High daily doses of pitavastatin have shown beneficial effects on therapeutic angiogenesis in experimental studies, but this regimen can lead to serious adverse effects in patients [127, 128]. To optimize the therapeutic effect of statins in inducing therapeutic neovascularization, Matsumoto, T. et al. constructed pitavastatin-incorporated poly (lactic-co-glycolic acid) NPs (NK-104-NPs) [129] and found no serious side effects after a single intramuscular injection in a mouse model of acute hindlimb ischemia [130]. Therefore, they conducted a phase I/II clinical trial [131] in 16 patients with CLI who received repeated intramuscular injections of NK-104-NPs containing 0.5, 1, 2, or 4 mg pitavastatin calcium for 5 days. They found NP-mediated increases in the local concentration and retention time of pitavastatin in ischemic tissues, which may be key determinants of the efficacy and safety of therapeutic neovascularization [131]. However, this approach requires larger placebo-controlled phase II/III clinical studies.

NPs can promote the targeted delivery of poorly water-soluble drugs to specific tissues or cell populations, thereby enhancing their efficacy. NP formulations can also be used to combine multiple therapeutic modalities at once, to prevent drugs from interacting with certain blood constituents and tissues to avoid toxicity, and to enhance the circulatory half-life and protect drugs from inactivation and degradation [132]. Also, the risk of oncogenic effects is low. Although NPs were shown to be safe in a clinical trial [131], they are exogenous substances and may elicit an immune rejection reaction [133], which could greatly affect the therapeutic effect. Therefore, surface modification of NPs is crucial to escape immune system recognition [134].

### Biomaterials

#### Polymers

With the vigorous development of biomaterial research, regenerative medicine strategies provide more possibilities for treating IVD. Hydrogel is an excellent biocompatibility material and an ideal candidate for delivering bioactive molecules and cells for therapeutic angiogenesis [135]. Li, C., et al. found that hyaluronic acid (HA) hydrogel with antioxidant capacity provided a prolonged release of VEGF. After injection into rat ischemic hindlimb muscles, HA hydrogels reduced lipid oxidation, regulated oxidative-related genes, and enhanced local blood flow in the muscle [136], which suggests that HA hydrogels have potential for treating limb ischemia.

Ferulic acid (FA) is a natural phenolic compound and has been shown to provide antioxidants and promote angiogenesis. Wang, C.Y., et al. revealed that a hydrogel providing a sustained release of FA effectively decreased venous injury in a mouse model of hindlimb ischemia caused by oxidative stress and improved blood flow [137]. The therapeutic effectiveness of gelatin hydrogel is being studied in clinical trials. In a randomized clinical trial, He, X. et al. [138] found that hydrogels are safe and feasible for stem cell delivery in CHD, which may improve the targeting of stem cell therapy. However, because this study did not have a hydrogel-only group, it is unknown whether the enhanced therapeutic effect came from the transplanted cells, the hydrogel itself, or a combination of the two. Moreover, MSC differentiation lineage could be affected by matrix stiffness [139], compression [140], and hydrostatic pressure [141]. These three influencing factors provide new ideas for manipulating cell fate and tissue regeneration, and these studies suggest that using hydrogels can help improve the therapeutic effect of other treatments.

#### Bioscaffold

MSCs have a therapeutically important role in promoting angiogenesis in CLI. However, more than 90% of the injected cell suspension is lost and does not engraft in cell therapy [142]. In recent years, tissue engineering using a 3D porous scaffold has been shown to improve cell engraftment by controlling cell attachment, providing mechanical support, and stimulating in vivo tissue growth [143]. Mu, R., et al. designed a glucomannan decanoate (GMDE) substrate mimicking fungal carbohydrates that highly and preferentially supported EC adhesion. They found that GMDE scaffolds effectively blocked endogenous galectin-1, which bridges ECs to the scaffolds, and promoted vascularization in a mouse limb ischemia model without delivering any exogenous pro-angiogenic factors [144]. This brings up the question as to what is the optimal combination of scaffold and cells that drives angiogenesis? More preclinical studies are warranted to address this issue. Svystonyuk, D.A. et al. [145] conducted a phase I clinical observational study to explore the clinical feasibility, safety, and effects of cell-free bioscaffold therapy in patients with CHD. They implanted bioscaffolds in 8 CHD patients. At up to 6 months after surgery, they found that the bioscaffold could redirect cardiac fibroblasts to rebuild the microvascular network and avoid tissue fibrosis. However, they were unable to quantify the degree of restoration of cardiac structure and function as a direct result of the observed reduction in fibrosis with a concomitant increase in angiogenesis or to identify interactive effects between these variables. More in vitro and preclinical experiments are required to explore the mechanisms involved. Norbert Frey et al. [146] found that IK-5001, an injectable bioabsorbable scaffold, can help restore left ventricular function in patients with CHD. However, the study lacked a randomized control group for comparison. They reported no adverse events, which may be due to the limited number of patients (*n* = 27). Larger follow-up studies are needed to confirm the safety and efficacy of the device.

In recent years, the potential therapeutic efficacy of nanomedicine and biomaterials in IVD has been explored (Table 5). Four nanomedicine and biomaterials–based therapeutic clinical trials in IVD all showed positive treatment outcomes as discussed in the above section (Table 6). The sustained release effect of biomaterials has become a unique highlight for therapeutic effects, which compensates for the shortcomings of other treatment methods to some extent. Similar to nanomedicine-based therapy, immune reactions, including excessive inflammation, scar formation, and immune rejection, may be a challenge in using biomaterials in the clinical setting [147–149]. Moreover, foreign body reactions to implants can greatly affect the safety and efficacy of these biomedical constructs [149]. In addition, the size of the nanomedicine affects the therapy. Polyion complex (PIC) micelles of 30-nm diameter and PIC vesicles of 100- and 200-nm diameter were shown to have different therapeutic effects in ischemic lesions in PAD [150]. These results reveal that controlling the size of nanomedicines is a promising study direction for developing novel angiogenic treatments in IVD.

**Table 5:** A summary of material (including nanomedicine and biomaterials) -based therapeutic preclinical studies in IVD

Nanomaterials/ biomaterials	Disease	Function	Research types	Ref
MSC-exosomes	ischemic diseases	Promote angiogenesis	Animal	[119]
Magnetic nanoparticles (consist of a Fe3O4 core and a silica shell)	CHD	Promote angiogenesis	Animal	[122]
HA hydrogel	PAD	Prolong the release of VEGF, reduce lipid oxidation and enhance local blood flow	Animal	[136]
FA-gel	PAD	Promote angiogenesis	Cellular and Animal	[137]
CS-NO	PAD	Promote angiogenesis	Cellular and Animal	[151]
PF hydrogel (VEGF and ANG-1 were incorporated)	CAD	Promote angiogenesis and provide mechanical support	Cellular and Animal	[152]

*ANG-1* Angiopoietin-1, *CAD* Carotid artery disease, *CHD* Coronary heart disease, *CS-NO* Nitric oxide-releasing chitosan hydrogel, *FA* Ferulic acid, *HA* Hyaluronic acid, *IVD* Ischemic vascular disease, *PAD* Peripheral artery disease, *PF* Polyethylene glycol-fibrinogen, *VEGF* Vascular endothelial growth factor

**Table 6:** A summary of material (including nanomedicine and biomaterials) -based therapeutic clinical trials in IVD

First author	Phase year	Disease	Treatment	Subject(n) treatment/control	Findings	Ref
Frey, N	Phase II 2014	CHD	Intracoronary delivery of an innovative, injectable bioabsorbable scaffold (IK-5001)	27/0	Proven feasibility and good tolerability	[146]
Florian, A	Phase II 2014	CHD	Intravenous iron-oxide administration	17/22	Improved infarct healing and a beneficial global left ventricular remodeling	[123]
Svystonyuk, D.A	Phase I 2020	CHD	Intramyocardial grafting collagen scaffold with HUCMSC	35/15	Proven safety and efficacy	[138]
Matsumoto, T	Phase I/IIa 2022	CLI	Intramuscular pitavastatin-incorporated nanoparticles	16/0	Proven safety and efficacy	[131]

*CHD* Coronary heart disease, *CLI* Critical limb ischemia, *FGF* Fibroblast growth factor, *HUCMSC* Human umbilical cord mesenchymal stem cell, *IVD* Ischemic vascular disease

## Conclusions

**Fig. 3:**
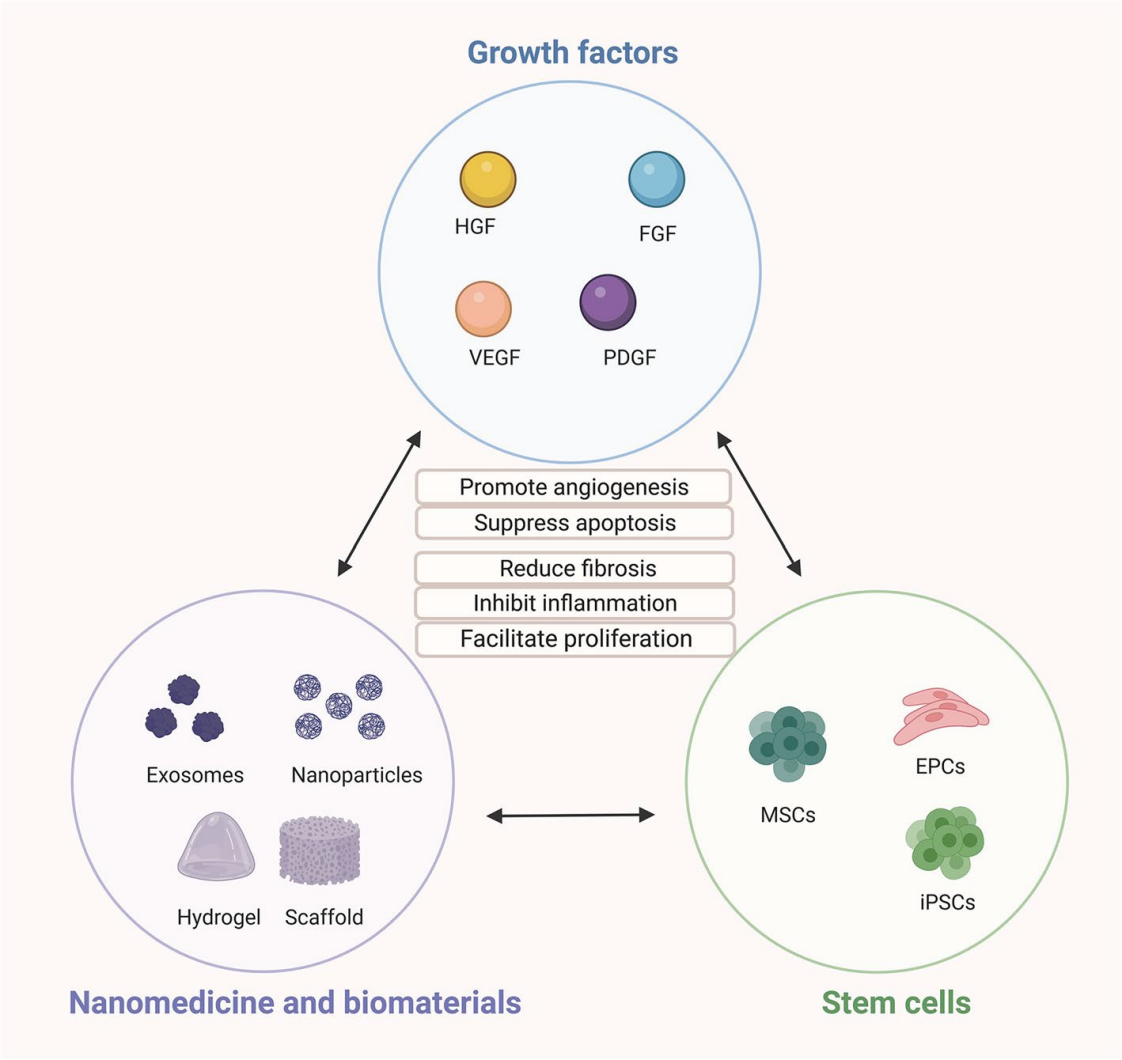
Three potential alternative therapeutics in ischemic vascular disease: growth factor-based therapy, cell-based therapy, and material-based therapy including nanomedicine and biomaterials. HGF: hepatocyte growth factor; FGF: fibroblast growth factor; PDGF: platelet-derived growth factor; VEGF: vascular endothelial growth factor; MSCs: mesenchymal stem cells; EPCs: endothelial progenitor cells; iPSCs: induced pluripotent stem cells. The figure was constructed with BioRender (https://biorender.com)

IVD is a common vascular disease, especially PAD and CAD. The main pathology involves damage to blood vessels, tissue ischemia, and downstream cellular events. Consequently, cell death and tissue damage ensue, depending largely on the degree and duration of the ischemic injury. For the heart, coronary blood flow is blocked, myocardial blood supply is insufficient, and myocardial diffuse fibrosis develops. Severe obstruction can lead to myocardial infarction. Brain ischemia can lead to swelling and necrosis of nerve cells, causing irreversible brain dysfunction. In recent years, therapeutic strategies around vascular regeneration and restoration of blood circulation have emerged. In this review, we have focused on the function, challenges, and future direction of three new promising treatment approaches: growth factor-based therapy, cell-based therapy, and material-based therapy including nanomedicine and biomaterials (Fig. 3). Analyzing these potential therapeutic strategies indicates that combining these three treatment methods may lead to a better IVD treatment effect. Rational designs that combine beneficial degradation products with the controlled release of therapeutic drugs inspire next-generation biomaterials designed to revolutionize regenerative medicine in IVD. The combination of the above three treatments is an important therapeutic strategy that deserves further study.

## Data Availability

Not applicable.

## Abbreviations

Ad-HGF: Adenovirus-hepatocyte growth factor
ADSCs: Adipose tissue-derived stem cells
ANG-1: Angiopoietin-1
bFGF: Basic fibroblast growth factor
BMSCs: Bone marrow mesenchymal stem cells
c-Met: Cellular-mesenchymal epithelial transition factor
CAD: Carotid artery disease
CHD: Coronary heart disease
CLI: Critical limb ischemia
CMs: Cardiomyocytes
ECs: Endothelial cells
EPCs: Endothelial progenitor cells
ER: Endoplasmic reticulum
ERK: Extracellular signal-regulated kinase
FA: Ferulic acid
FGF: Fibroblast growth factor
GDF: Growth differentiation factor
GMDE: Glucomannan decanoate
HA: Hyaluronic acid
HGF: Hepatocyte growth factor
HUCMSCs: Human umbilical cord mesenchymal stem cells
IGF: Insulin-like growth factors
IL: Interleukin
iPSCs: Induced pluripotent stem cells
IVD: Ischemic vascular disease
LAD: Left anterior descending artery
LVEF: Left ventricular ejection fraction
MAP: Mitogen-activated protein
MAPK: Mitogen-activated protein kinases
MI: Myocardial infarction
microRNA: Micro ribonucleic acid
mRNA: Messenger ribonucleic acid
MSC: Mesenchymal stem cell
mTOR: Mammalian target of rapamycin
NPs: Nanoparticles
PAD: Peripheral artery disease
PDGF: Platelet-derived growth factor
PF: Polyethylene glycol-fibrinogen
PI3K: Phosphoinositide-3 kinase
PIC: Polyion complex
PlGF: Placental growth factor
PR1P: Prominin-1-derived peptide
ROS: Reactive oxygen species
VEGF: Vascular endothelial growth factor
